# The relationship between periodontitis and proteinuria in chronic kidney disease: A review

**DOI:** 10.4317/medoral.27304

**Published:** 2025-05-27

**Authors:** Guangxun Zhu, Lili Chen, Qian Liu

**Affiliations:** 1 Department of Stomatology, Tongji Hospital, Tongji Medical College, Huazhong University of Science and Technology, Wuhan, China; 2 School of Stomatology, Tongji Medical College, Huazhong University of Science and Technology, Wuhan, China; 3 Hubei Province Key Laboratory of Oral and Maxillofacial Development and Regeneration, Wuhan, China; 4 Department of Stomatology, Union Hospital, Tongji Medical College, Huazhong University of Science and Technology, Wuhan, China

## Abstract

**Background:**

Proteinuria is elevated protein in the urine possibly progressing to glomerular sclerosis, which was frequently observed in chronic kidney disease (CKD), diabetes, preeclampsia, etc. Previous studies have revealed that periodontitis and these diseases share common risk factors, so a study is necessary to discuss the potential relationship between periodontitis and proteinuria. For the moment, there are no reports that are concerned about the correlation between periodontitis and proteinuria.

**Material and Methods:**

We searched PubMed for studies associated with periodontitis and proteinuria published before March 2025.

**Results:**

Existing evidence showed that periodontitis might increase the risk of proteinuria, as periodontal pathogens and periodontal inflammatory reactions were proven to injure the glomerulus and renal tubules contributing to the progression of proteinuria. On the other way, proteinuria might affect systemic inflammation and bone metabolism to increase the risk of periodontitis.

**Conclusions:**

This article reviews the relationship between periodontitis and proteinuria, reveals their predicTable potential for chronic kidney injury, and makes recommendations for the treatment of periodontitis and proteinuria.

** Key words:**Proteinuria, albuminuria, periodontitis, inflammation, renal disease.

## Introduction

Periodontitis is mainly caused by the interaction of the pathogenic subgingival plaque biofilm and its virulent products with the periodontal tissues of the host, which increases inflammatory cytokines levels both in the local tissue and the circulatory system. Emerging evidence has indicated that periodontitis has a bidirectional relationship with several diseases including chronic kidney disease (CKD), diabetes, hypertension, liver disease, adverse pregnancy outcomes, atherosclerosis, and even cancers (1). The main association between periodontitis and these diseases is the systemic low-grade endoxemia and low-grade inflammation. All these diseases can induce an inflammatory response and create inflammatory mediators to influence each other. Markedly elevated plasma endotoxin levels were found in CKD patients. Low-grade inflammation e.g. serum IL-6 and C-reactive protein (CRP) was associated with kidney function decline, and thus is of relevance to periodontitis.

Proteinuria is the presence of protein, usually albumin, in urine, which can be caused by dehydration, emotional stress, strenuous exercise, and certain medications in benign conditions. It also signifies a primary nephropathy or a disease secondary to diabetes, heart failure, preeclampsia, etc. Proteinuria is mainly caused by the increased permeability of glomeruli and their subsequent weakened reabsorption by the proximal tubular epithelium. Impaired immune responses and genetic factors as well as microbiota could cause the pathogenesis of proteinuria by impacting many aspects of the innate and adaptive immune systems. Lipopolysaccharide (LPS) was reported to induce massive proteinuria by the fusion of glomerular podocytes with foot processes. LPS would directly stimulate the Toll-like receptor-4 (TLR-4)/CD14 receptor and rapidly upregulate CD80 in podocytes *in vivo*, leading to proteinuria. On the contrary, LPS removal was reported to reduce proteinuria. Our previous work reviewed the potential association between gingival bleeding and haematuria in renal diseases (2). Haematuria and proteinuria in chronic kidney disease (CKD) are both signals of glomerular dysfunction and are routinely detected via urinalysis and serve as critical tools for early CKD diagnosis and treatment assessment. Furthermore, proteinuria is a more well-known risk factor for progression to CKD in adults and children. Besides, proteinuria was an independent predictor of both cardiovascular and kidney outcomes in CKD. Currently, both proteinuria and periodontitis are considered in connection with systemic inflammation and microbiota, so a positive correlation is hypothesized between periodontitis and proteinuria. This review aimed to elicit the relationship between periodontitis and proteinuria and reveal their predicTable potential for chronic kidney injury.

-Proteinuria

Proteinuria indicates an elevated concentration of protein in the urine (normal excretion should be < 150 mg/d), which contains low-molecular-weight proteins e.g., α1-microglobulin and β2-microglobulin, intermediate-molecular-weight protein e.g., albumin, and high-molecular-weight proteins e.g., IgG and IgM, etc. The prevalence of proteinuria was reported at 8.45%, 11.5%, and 23.6% in the general population in Croatia, British, and sub-Saharan Africa, respectively (3). The primary etiology of proteinuria is the disturbance of the kidney filter. Proteinuria is classified to be transient and persistent. Transient proteinuria is observed in urinary tract infections, fever, heavy exercise, vaginal mucus, etc. However, persistent proteinuria is a sign of kidney damage. The diagnosis of criteria for CKD includes proteinuria >200mg/day or protein to creatinine ratio (PCR) >200mg/g or albuminuria (urinary albumin excretion (UAE) ≥30mg/day or albumin to creatinine ratio (ACR) ≥30mg/g. Furthermore, it also occurs in the condition of renal injury secondary to diabetes, hypertension, and other CV disease. Mostly, the degree of proteinuria coordinates with disease progression.

Dip-stick proteinuria is the most frequently used method to measure proteinuria and screen for CKD. The National Kidney Foundation recommended urine protein concentrations of 30-99 mg/dL, 100-299 mg/dL, 300-999 mg/dL, and ≥ 1000 mg/dL to be labeled as “1 + ,” “2 + ,” “3 + ” and “4 + ,” respectively. However, it is only sensitive to albumin, inevitably exhibiting a low sensitivity and low specificity in detecting proteinuria. A 24-hour urine protein test, the amount of albumin in urine over 24 hours, is also commonly used and more cumbersome to monitor the health of patients suspicious of kidney problems. The result between 30 and 300 mg/24h is called albuminuria (or microalbuminuria), and above 300 mg/24h - proteinuria. Urinary Albumin Excretion (UAE) refers to the measurement of albumin (a protein normally retained by healthy kidneys) excreted in urine over a specific period. UACR (Urinary Albumin-to-Creatinine Ratio) assesses the amount of albumin (a type of protein) excreted in urine relative to creatinine (a waste product from muscle metabolism) and is calculated by dividing the concentration of urinary albumin (in milligrams) by the concentration of creatinine (in grams) in the same urine sample. Albuminuria means “abnormal loss of albumin in the urine.” It was classified into two categories: microalbuminuria (UAE = 30-300 mg/day or ACR = 30-300 mg/g creatinine) and macroalbuminuria (UAE > 300 mg/day or ACR > 300 mg/g creatinine). The nephron governs the urinary protein excretion from two aspects: one at the glomerular filtration level and the other downstream of proximal tubular cell uptake to degrade the filtered albumin. Hence, there are two common reasons for proteinuria as follows:

One main reason is increased permeability of the glomerular capillary wall. Glomerular capillary endothelial cells, podocyte, and glomerular basement membrane (GBM) constitute the glomerular mechanical barrier and charge barrier to prevent the exudation of proteins and cells from the glomeruli. The impairment of the GBM charge selectivity and the damaged glomerular pore size in the capillary wall would permit the unrestricted transglomerular passage of albumin, which is characteristically found in the initial stages of many glomerular diseases.

Podocytes would sense LPS, glomerular hypertension/hyperfiltration, and reactive oxygen species, and then release inflammatory cytokines and recruit immune cells into the glomerulus. Excessive stimulation could cause podocyte injury, which signifies the critical beginning of proteinuria. Slit membrane molecules, cell adhesion molecules, and the actin cytoskeleton in podocytes form a firm network to implement the selective permeability function of GBM. Abnormalities of podocyte actin systems lead to foot process effacement in minimal change disease. Persistent or severe stimulation leads to podocyte cell detachment, promoting further glomerular damage.

Glomerular endothelial cells, the inner layer of the glomerular capillary wall, are covered with a carbohydrate-rich glycoprotein called the glycocalyx. The endothelial glycocalyx is anchored to the endothelium through membrane-bound proteoglycans and possesses the ability to bind soluble albumin. Disruption of glomerular endothelial glycocalyx increases the microvascular permeability and coincides with albuminuria in glomerular disease. Furthermore, the inflammation state would cause degradation of glycocalyx which is mediated by matrix metalloproteinases, reactive oxygen species, tumor necrosis factor (TNF)-α, heparinase, and thrombin.

Besides, endothelial cell injury manifests increased permeability to albumin. The thickening of the GBM may reduce cell binding and promote cellular detachment to promote proteinuria. Each of these layers within the glomerular barrier establishes its unique function to prevent the occurrence of proteinuria in the overall GBM permeability. Injure to any layer would develop proteinuria and promote glomerular sclerosis.

The second reason is impaired reabsorption by the epithelial cells of the proximal tubules. Normally, the filtered low-molecular-weight protein is reabsorbed by the epithelial cells of proximal tubules. When the increased permeability of the glomerular capillary wall occurred, the reabsorption was saturated, then the filtered proteins appeared in the urine. Furthermore, the epithelial cells of the proximal tubules, exposed to high-dose albumin, may progressively lose their integrity. The impairment of lysosomal function and morphologic changes by the upregulated gene expression and production of inflammatory mediators occurs in these renal tubular epithelial cells. Accumulating evidence suggests that several proinflammatory cytokines would lead to an inflammatory and fibrotic response in the renal interstitium (4). These filtered proteins would cause the release of inflammatory and fibrogenic mediators, resulting in the recruitment of mononuclear cells into the interstitium. Then this phenomenon would cause fibroblast proliferation and extracellular matrix accumulation, pathologically manifesting tubular atrophy and interstitial fibrosis. The mechanisms underlying proteinuria, glomerular sclerosis, and tubulointerstitial fibrosis overlap partially, which need extensive efforts to clarify their relationship.

Also, secretory proteinuria means the oversecretion of specific proteins in the tubules. Meanwhile, excessive protein secretion can saturate the reabsorptive abilities of the proximal tubules, leading to overflow proteinuria.

-Periodontitis

**Figure 1:**
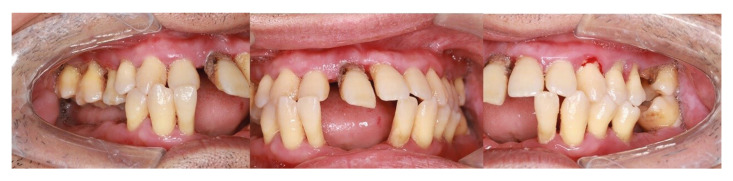
Intraoral photographs of CKD patients. Chinese, male, 51 years old, non-smoker, manifesting teeth loss, gingival bleeding, deep probing depth, and gingival recession.

Periodontitis, the sixth most common non-communicable human disease, is the inflammation of periodontal tissue with a high prevalence of 80%-90% in adults over 35 of age. Periodontal loss is determined by the aberrant immune reaction of periodontal tissue and subgingival microbial communities dysbiosis. The prevalence of CKD was estimated at 9.1% in the world's population in 2017. China had the most CKD patients (132.3 million cases), followed closely by India (115.1 million cases). Bangladesh, Brazil, Indonesia, Japan, Mexico, Nigeria, Pakistan, Russia, the USA, and Vietnam had more than 10 million cases of CKD each (5). Uremic toxins accumulation in CKD patients could stimulate various unbalanced cytokines and inflammation-related molecule production including increased CRP and upregulated expression of intercellular adhesion molecule-1 (ICAM-1), leading to aberrant inflammatory reactions. Bacterial dysbiosis with an inclination to periodontal disease was also shown in CKD. Notably, CKD influences the periodontal bone metabolism process. The biochemical parameters of mineral bone disorders including increased fibroblast growth factor 23 (FGF23), hyperphosphatemia, hypocalcemia, elevated parathormone (PTH), and 1,25-dihydroxy vitamin D3 deficiency, were frequently observed in CKD (6). A systemic analysis found that CKD was related to a higher risk of periodontitis, manifesting higher clinical attachment loss and higher probing depth compared to healthy individuals (7). As Fig. 1 shows, CKD patients exhibited more severe periodontal destruction, e.g. teeth loss, gingival bleeding, deep probing depth, and gingival recession. Hence, periodontitis would be a frequent CKD comorbidity.

On the other way, periodontitis is associated with various systemic diseases involved with the gastrointestinal, respiratory, cardiovascular, and urinary systems of the body. The main mechanisms for periodontitis exacerbating inflammation in distant tissues contain systemic bacteremia, cytokine release, and inflammation. Our former research has detailed introduced that those periodontal pathogens stimulated IgA deposition in their glomerular basement membrane and injured the endothelial cells of the glomerular capillary wall (2). Periodontitis could be a permanent source of low-grade inflammation to influence the kidney's immune response. Furthermore, periodontitis increases the progression of kidney dysfunction and deteriorates the outcomes of these patients. An increasing number of evidence from epidemiological and clinical observations exhibits that periodontitis increases the morbidity and mortality of CKD (4).

Oral microbiota dysbiosis is the main cause of periodontitis. Until now, the direct evidence of changes in oral microflora in CKD is not sufficient. However, it brought about widespread attention that gut microbiota contributed to the pathogenesis of CKD in recent years. Indirectly, gut microbiota could be disrupted by orally derived periodontal pathogenic bacteria (8).

Periodontal pathogens and their byproducts could disseminate from the pocket depth to the blood circulation, inducing a systemic inflammation with an extra-oral metastatic infection. LPS could mediate TLR activation through My88 normally results in NF-kB-mediated transcription of proinflammatory cytokines, and recruitment of inflammatory cells of the adaptative immunity in periodontal tissue and kidney tissue. Indeed, the presence of bacteria and LPS induces the host secretion inside the bloodstream of pro-inflammatory cytokines (TNF-α, IL-6, CRP, oxygen radicals) involved in the increase of systemic inflammatory state. Accordingly, periodontal non-surgical treatment leads to a significant decrease in systemic inflammation biomarkers.

The mechanistic link between periodontal disease and kidney diseases also contained inefficiency of the immune system, caused by persistent low-grade inflammation in periodontitis and CKD. Impaired innate and adaptive immunity was also found in periodontal tissue and kidney tissue. In CKD, decreased capacities of monocyte stimulation and neutrophil phagocytosis were observed in kidney tissue (6). Diminished TLR4 expression was associated with reduced synthesis of TNF-α, IL-6, and IL-1β in response to LPS challenge in CKD patients. Impaired immunity including a deregulated complement system, immune complex deposition, glomerular damage due to autoantibodies, and imbalance between regulatory T cells and effector T cell subsets would cause glomerulonephritis, which was an important cause of CKD.

Periodontitis also influences endothelial functions including active regulatory functions on the balance of oxidative stress, vasoconstriction/vasodilation, platelet aggregation, and leukocyte adhesion (9). Meanwhile, endothelial dysfunction was an important etiology of CKD and subsequently declines glomerular filtration.

The incidence of periodontitis can influence kidney function, tissue, and damage. Periodontitis was reported to have a certain relationship with hematuria, glomerular filtrate rate, and CKD (7). Even more, there was a certain link between the severity of periodontitis and kidney damage. However, the association between periodontitis and proteinuria was unclear.

## Material and Methods

-Study about periodontitis and proteinuria

An online bibliographic retrieval was searched in PubMed until 2025.3 to reveal the relationship between periodontitis and proteinuria. A series of MeSH term words (periodontal, periodontium, periodontitis, gingival, gingiva, and gingivitis) with proteinuria and albuminuria were searched. The initial literature search identified 63 citations. 28 records were removed after the abstract screening. 7 records were excluded as review. 17 records were excluded for not reporting relevant outcomes. Finally, 11 articles satisfy the assessing standards in the retrieved literature. As proteinuria is the hallmark of both primary and secondary proteinuric glomerulopathy, the association between periodontitis and proteinuria was found in primary kidney injury, diabetes, hypertension, and preeclampsia. Statistical analysis was performed using the software RevMan 5.4.

## Results

-Periodontitis increased the risk of proteinuria in the general population, kidney disease, diabetes, and increased the risk of preeclampsia

Proteinuria is an important symptom of kidney dysfunction. Another study reported that severe periodontal destruction exhibited more severe kidney pathological change and higher levels of 24-hour proteinuria in 436 IgA nephropathy hospitalized patients in the Department of Nephrology of China-Japan Friendship Hospital (10).

The incidence of proteinuria in the general population is about 8%~33%. In the general population, a study explored the relationship between CPI scores and low-degree albuminuria of 23,626 participants from the Korean National Health and Nutrition Examination. Macro-albuminuria (UACR≥30 mg/g) and diabetes were excluded. Finally, 11,617 non-diabetes adults were investigated at Yeungnam University Hospital. The results showed the UACR values were significantly higher in participants with periodontitis (38.8±1.2%) than in those without periodontitis (27.8±0.7%). Periodontitis was proved associated with a high UACR [OR and 95% CI: 1.89 (1.04-3.42)] (11). Another follow-up study showed that periodontal pocket depth (PPD) is positively associated with ACR (r = 0.12; *P* <.01), fasting blood glucose (r = 0.28; *P* <.01), and hemoglobin A1c (HbA1C) (r = 0.26; *P* <.01) among 1486 patients with CKD and periodontal diseases (12). Combined PPD and HbA1C were reported to be a valuable predictor of CKD progression in patients with periodontal diseases. Tsai *et al* investigated the prevalence of proteinuria and periodontitis in a total of 1280 participants aged 18-45 years old at the Hualien Armed Forces General Hospital between 2018 and 2021. There was a correlation between localized stage II/III periodontitis (incidence: 57.6%) and dipstick proteinuria 2+ and 3+ (incidence: 10.8%) [odds ratio (OR) and 95% confidence interval (CI): 1.89 (1.04-3.42)]. However, there was no significant association between periodontitis and stage 2 CKD (13). On the contrary, a study reported periodontitis increased the risk of low GFR but not with incident albuminuria (ACR ≥30 mg/g) in CKD (14), which might be attributed to inappropriate grouping.

Poorly controlled diabetes can lead to injury of blood vessel clusters in the kidneys, resulting in the onset of proteinuria. Many longitudinal studies for diabetes patients reported that severe periodontitis cases exhibited a higher prevalence of albuminuria, cardiovascular complications, and end-stage renal disease (ESRD) (15-17). 529 diabetes individuals were followed up for 22 years in the Gila River Indian Community of Arizona. The result showed that age- and sex-adjusted incidence of macroalbuminuria was increased with the degree of periodontitis destruction. The incidence of macroalbuminuria was 2.0, 2.1, and 2.6 times higher in individuals with moderate or severe periodontitis or edentulous jaw than those with none/mild periodontitis. The incidence of ESRD in the moderate/severe-periodontitis group was 2.3, 3.5, and 4.9 times higher than the none/mild-periodontitis group (16).

After diabetes, hypertension is another secondary cause of proteinuria. That is because proteinuria is affected by altered glomerular hemodynamics and hyperfiltration. Tsioufis *et al* found that periodontal indexes and CRP have a synergistic effect on UACR levels in untreated middle-aged hypertensive patients. Furthermore, this synergistic effect is independent of the underlying hemodynamic load resulting from hypertension (18). Preeclampsia is a hypertensive disorder (140/90 mmHg) in pregnancy, which manifests high protein levels in urine (2+ proteinuria) and at least 0.3 g proteinuria/24 hours. One meta-analysis of 11 studies involving 1118 women with preeclampsia and 2798 women without preeclampsia reported that the risk of preeclampsia is 3.69-fold higher in women with periodontal disease before 32 weeks of gestation than their counterparts without periodontal disease (OR=3.69; 95% CI: 2.58-5.27) (19). Another meta-analysis involving overall 3420 women (493 preeclamptic and 2927 non-preeclamptic control women) reported that the risk of preeclampsia is 1.76-fold higher in women with periodontal disease during pregnancy than women without periodontal disease (OR, 1.76, 95% CI: 1.43-2.18) (20). In one study, Contreras compared the periodontal status of 130 preeclamptic and 243 non-preeclamptic women in Colombia. He concluded the preeclamptic group showed significantly higher clinical attachment loss than the non-preeclamptic group. Meanwhile, the red complex microorganisms, the pathogenic bacteria of periodontitis, were more frequently observed in the preeclamptic group. Hence, the author concluded that periodontitis would cause the pregnant woman to develop preeclamptic (21).

**Figure 2:**
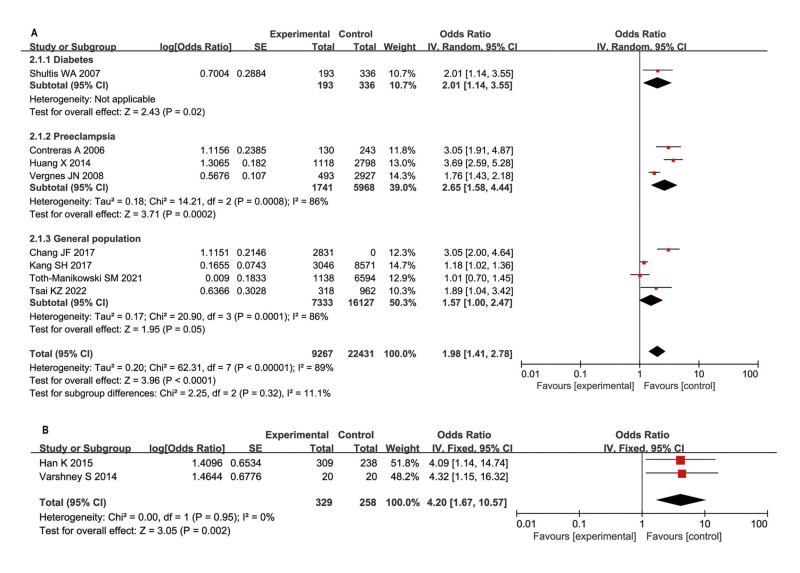
ORs and 95% CIs from the included studies on the association of periodontitis and proteinuria in random-effects models (A. periodontitis increased the risk of proteinuria; B. proteinuria increased the risk of periodontitis).

Hence, we could conclude that periodontitis increased the risk of proteinuria in the general population, kidney disease, diabetes, and increased the risk of preeclampsia. For statistical analysis, eight studies (11-14,16,19-21) reported the association between periodontal disease and the risk of proteinuria in a random-effects model (OR: 1.98; 95% CI: 1.41-2.78). For subgroup analysis, periodontitis increased the risk of macroalbuminuria in diabetes patients (OR: 2.01; 95% CI: 1.14-3.55). Periodontitis in pregnant women might develop preeclampsia (OR: 2.65; 95% CI: 1.58-4.44). In the general population, periodontitis increased the risk of proteinuria (OR: 1.57; 95% CI: 1.00-2.47) (Fig. 2).

-Proteinuria increases the risk of periodontitis in CKD, diabetes, and preeclampsia.

Periodontitis, a frequent CKD comorbidity, showed consistency of severity with CKD. The degree of proteinuria correlates with CKD progression, either. Hence, we concluded that the extent of proteinuria might coordinate with the extent of periodontitis in CKD. The causation that proteinuria increases the risk of periodontitis was a parallel in relationship with chronic inflammation of proteinuria and periodontitis (7). In a study of 547 patients with type 2 diabetes without renal impairment in Korea, the patients with albuminuria showed a significantly higher prevalence of periodontitis (UACR >30 mg/g), compared with those patients without albuminuria. Hence, the author concluded the risk of periodontitis had a positive correlation with proteinuria in adults with type 2 diabetes (22). In another study, 503 patients with type 2 diabetes were enrolled in the Jiyugaoka Medical Clinic in 2015. Participants with albuminuria had significantly higher % sites of pocket depth ≥ 4 mm, elevated HbA1c, and upregulated CRP, compared with subjects without albuminuria. The results also showed significant associations between albuminuria and several periodontal parameters (23). Another study showed that patients in the case of albuminuria had significantly fewer teeth left in type 2 diabetes mellitus patients with chronic periodontitis (17). A case-control study including 40 pregnant women patients in J.N. Medical College showed that the preeclamptic cases had 4.33 times higher risk of having periodontal disease than non-preeclamptic cases (24). For statistical analysis, two studies (22,24) reported the association between proteinuria and the risk of periodontitis involving 329 proteinuria patients and 258 non-proteinuria patients in a random-effects model (OR: 4.20; 95% CI: 1.67-10.57) (Fig. 2).

Disscussion

-Possible mechanistic relationship between periodontitis and proteinuria in CKD

**Figure 3:**
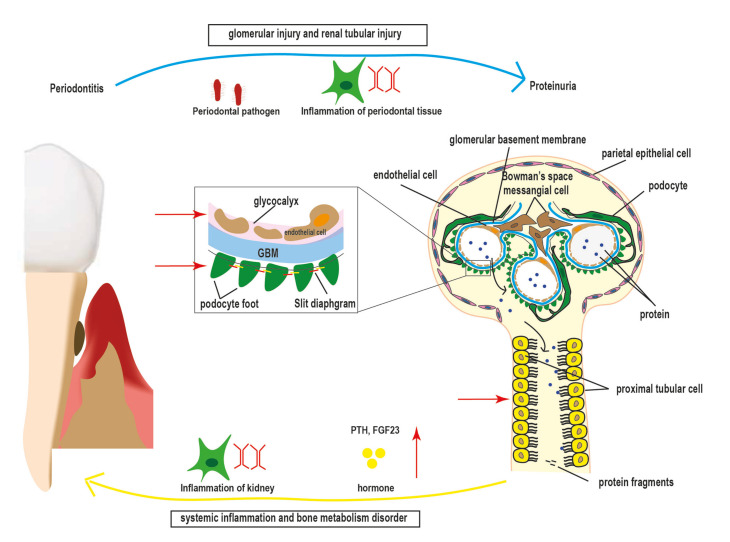
The relationship between periodontitis with proteinuria in CKD patients. Periodontitis increases the risk of proteinuria by glomerular injury and renal tubular injury. Furthermore, periodontal pathogen and inflammation of periodontal tissue might damage the podocyte and glycocalyx to injure glomerular filtration. They might hurt proximal tubular cells resulting in renal tubular injury. Proteinuria increases the risk of periodontitis by systemic inflammation and bone metabolism disorder. Proteinuria contributes to the inflammation of the kidney, and elevated hormones e.g. PTH, and FGF23, result in severe periodontal loss.(GBM) glomerular basement membrane; (FGF) Fibroblast Growth Factor; (PTH) Parathyroid Hormone.

Above all, we have hypothesized that periodontitis may have a relationship with proteinuria: 1) Mechanistic link between periodontitis and proteinuria; 2) Periodontitis and proteinuria are both the risk factors of CKD, CV, and mortality; 3) periodontal therapy would relieve or eliminate proteinuria. This association is shown in Fig. 3.

Generally speaking, proteinuria reveals microvascular injury to the kidney and can be a clinical sign of renal injury due to chronic inflammation. Periodontitis is associated with an imbalance of an inflammation response including immune function, neutrophil activity, and the production of various cytokines. Therefore, both periodontitis and proteinuria are recognized as a maker of chronic inflammation that promotes systemic inflammation and is related to vascular endothelial dysfunction . They are shown to be interrelated and deleteriously affect each other. Hence, the mechanistic link between periodontitis and proteinuria is the chronic inflammation originating from the oral cavity and kidney.

Periodontitis is related to glomerular injury.Various excessive stresses and pathological stimuli like oxidative stress (LPS, ischemia-reperfusion, chemical/toxic substances), immunologic stress, and mechanical stress (glomerular hypertension/hyperfiltration) could lead to stress-maladaptation with complex biological reactions including integrity loss and cell metabolic disorders of the podocyte, endothelial cells, and leukocytes.

Then detachment of podocyte from GBM occurs. Protein disrupts endothelial glycocalyx and GBM. Patients with severe periodontitis tend to suffer from bacteremia origin of the mouth, leading to the renal accumulation of bacterial elements. *Campylobacter* rectus, a periodontal pathogen prevalent in IgA nephropathy patients by analysis of saliva samples, was associated with proteinuria (2). When P.gingivalis LPS was administered within a streptozotocin-induced diabetic mouse, the kidney tissue images showed the upregulated IL-6, upregulated transforming growth factor (TGF) -β expression, and accumulated type 1 collagen in glomeruli (25). Another researcher found that P.gingivalis LPS would induce overexpression of leukocyte adhesion molecules to induce the infiltration of Mac-1/podoplanin-positive macrophages in glomeruli (26). Accumulated data suggested hyperactivated macrophages would induce podocyte apoptosis and promote podocyte foot process effacement and actin rearrangement, contributing to proteinuria.

Disruption to the endothelial glycocalyx coincides with albuminuria, which has been detected in numerous physiological and pathological conditions. Endothelial cell dysfunction would generate oxidative stress to activate sheddase ADAM-17 to degrade glycocalyx. Endothelial dysfunction also showed increased permeability to albumin. Hence, increased degradation of glycocalyx is closely related to endothelial cell dysfunction. A lot of clinical evidence and epidemiological observation have explicitly introduced the correlation between periodontitis and endothelial dysfunction, which might cause proteinuria. Intensive periodontal treatment has significantly improved endothelial function in parallel with the improvement in periodontal variables. Furthermore, P.gingivalis LPS promoted upregulated toll-like receptor 2 and 4 expressions in the endothelium in diabetic mice, and induced cytokines production from the endothelium. The repeated administration of LPS accelerated the progression of proteinuria and caused the diabetic mice to die within the survival period (9).

An influx of immune complexes and inflammation mediators produced by the periodontal lesion, e.g. antibodies, IL-1β, IL-6, and TNF-α, can enter into the systemic circulation and distant organs such as the kidney. One cross-sectional study investigated the association between serum antibodies to P.gingivalis and CKD in 215 elderly Japanese individuals. The result showed that increased serum antibodies to P.gingivalis significantly correlated with decreased kidney function (27). Nephrin is a key transmembrane protein to maintain normal slit diaphragm structure in podocytes. Increased serum TNF-α downregulated nephrin expression in the glomeruli in diabetic nephropathy (28). High-level serum TNF-α also had an impact on renal vascular endothelial growth factor (VEGF)/endothelial nitric oxide synthase (eNOS) expression, which is critical for normal vascular endothelial function and glomerular capillary wall homeostasis (29). We could infer that the circulation immune complex produced by periodontitis might lead to microvascular abnormalities and podocyte injury to increase proteinuria.

Chen *et al* reported that periodontitis caused glomerular volume enlargement, glomerular structure anomalies, reduced Bowman’s space, and glomerulosclerosis in obese mice (30). Hence, periodontal pathogen and periodontal inflammation influence glomerular tissue, function, and damage, resulting in proteinuria.

Meanwhile, Periodontitis is related to renal tubular injury. Periodontitis was reported to aggravate renal tubular structural anomalies manifesting vacuolar degeneration, loss of the brush border and tubular dilatation, increased exfoliation of the epithelial cells lining tubules, and movement of several nuclei from the basement membrane to the lumen. Plaque control would improve the histological change of renal tubular structure. Cytokine production of periodontal origin also made renal tubular cells swell into vacuoles and exhibited tubular luminal dilatation in kidney tissue (30). P.gingivalis LPS also promoted the accumulation of unmetabolized renal-specific hormones and enzymes e.g., osteocyte-derived hormone FGF23 in blood and kidney, resulting in hypertension. The FGF23 could promote phosphate excretion by directly targeting proximal tubules. Hence, we could infer that periodontitis irritates renal tubulitis. Increased unmetabolized FGF23 in the kidney and blood would provide a prediction of proteinuria in diabetic patients with periodontitis (26). Meanwhile, P.gingivalis LPS stimulation caused elevated glucose reabsorption by excessive production of sodium-glucose cotransporter 2 (SGLT2) in renal tubular structure in diabetic nephropathy. The histological results showed strong SGLT2 expression at the tubular wall around blood vessels. Abnormally high SGLT2 was also frequently observed in the outer wall of the tubules and the renal proximal tubular lumen. These results implied that the inflammatory response stimulated by P.gingivalis LPS exacerbated proteinuria by accelerating diabetic nephropathy progression (31).

Besides, proteinuria influences periodontitis. Accumulating evidence introduced that both proteinuria and periodontitis had systemic effects on the body such as chronic inflammation and dysfunction of immune cells. The systemic immune-inflammation index (SII) is an index to reflect the local and systemic immune response and inflammation in the whole human body. It is calculated by platelet count × neutrophil count/lymphocyte count. A total of 36,463 individuals were enrolled and examined for SII and urinary protein excretion through the National Health and Nutrition Examination Survey (NHANES). The results showed that SII correlated with the degree of proteinuria (32). Meanwhile, another multicentered, double-blind, hospital-based case-control clinical study reported that SII in patients with generalized stage III grade C periodontitis was significantly higher than periodontally healthy individuals (*p* < 0.0001) (33). Hence, systemic immune inflammation was an important link between proteinuria and periodontitis.

Glomerular injury and tubular injury would induce hyperphosphatemia, which in turn regulates PTH, FGF23, vitamin D, etc. In a podocyte-specific injury mice model manifesting strong proteinuria, serum Pi, serum FGF23 and intact PTH increased gradually, which might lead to secondary hyperparathyroidism and bone disease (6). Meanwhile, a cross-sectional study showed that higher albuminuria was reported to be associated with lower bone mineral density in diabetes, which in turn influences the process of periodontal bone remodeling (34). Another research reported that higher serum PTH might cause CKD animals to exhibit lower mandible bone volume in the region of the first mandible molar and a phenotype consistent with periodontitis. This result was consistent with a small cohort of dialysis patients showing alterations in facial bones including the mandible where PTH is elevated. Proteinuria is also reported associated with increased circulation levels of FGF23, a core regulator of phosphate metabolism. A multivariable regression analysis of CKD patients showed that proteinuria was significantly and independently associated with elevated FGF23 levels when taking blood pressure, endothelial dysfunction, eGRF, and hs-CRP into account (35). However, FGF23 is also involved in the inflammation process and bone mineralization homeostasis, which is important for maintaining healthy teeth and skeletons. Hence, we concluded that proteinuria would increase the risk of periodontitis by hormone change.

Periodontitis and proteinuria are both risk factors for CKD, CV, and mortality. Periodontitis and proteinuria share several common risk factors including male gender, older age, overweight/obesity, smoking, diabetes, and low socioeconomic status in several different populations. Statistically, numerous research revealed that albuminuria significantly increased the risk of mortality in the general population, patients with heart failure, and patients with CKD. Patients with proteinuria had a significantly higher risk of major adverse cardiovascular events in CV patients, independently associated with a worse outcome. Indeed, severe periodontitis was reported to be a significant and independent risk factor of CKD, CV, and all-cause mortality, either. Periodontal conditions including poor oral hygiene, deep probing depth, and severe attachment loss showed a higher risk of coronary heart disease. Therefore, periodontitis and proteinuria are both risk factors for CKD, CV, and mortality.

Periodontal treatment would lead to remission of proteinuria. Anti-inflammation therapy would alleviate the progression of proteinuria. Effective receptor antagonists targeting pro-inflammatory macrophages were shown to reduce renal injury and proteinuria in many clinical trials. Furthermore, periodontal treatment would decrease serum IL-1, and IL-6 to reduce proteinuria. An interventional clinical trial enrolled 50 sTable subjects of CKD (stage III-IV) and chronic periodontitis. The experimental group received periodontal treatment and showed a significant difference in periodontal parameters, IL-1β, and CRP, compared with the control group who did not receive periodontal treatment (36). A cohort study enrolled 10 patients with primary glomerulonephritis and interfered with appropriate dental treatments. The results showed that periodontal therapy decreased the median urine protein excretion (37). Another pilot study reported that 12 participants with diabetes were given oral hygiene instructions and subsequent scaling and root planning. It showed that the levels of urinary N-acetyl-β-D-glucosaminidase and albumin decreased significantly after treatment (38). Another study enrolled 80 CKD patients aged 22-65 years to accept periodontal treatment. The significantly decreased UACR and deceased periodontal parameter scores, and the decreased serum levels of CRP were observed in the patients who accepted the non-surgical periodontal treatment 3 months and 6 months later (*P* < 0.001) (39). The aforementioned research proved that periodontal treatment might promote remission of proteinuria. However, these clinical trials were not multicentered, double-blind, large-sample, randomized controlled trials to fully determine the efficacy of periodontal therapy on proteinuria. Bias could not be avoided including selection bias, performance bias, detection bias, and reporting bias in the above research.

## Conclusions

Above all, periodontitis and proteinuria have a certain relation. Multi-level studies, including clinical evidence, cellular biology research, and animal experiments, have demonstrated a significant association between proteinuria and periodontitis. The underlying mechanism might be a conjunct immune-inflammatory response origin from periodontitis and proteinuria (see Fig. 3).

In this regard, the specific mechanism of the bidirectional correlation between periodontitis and proteinuria should be investigated in future studies. Multicentered, double-blind, randomized controlled trials are needed to confirm the outcome of periodontal therapy on the remission of proteinuria. Finally, these results would provide evidence-based advice for reducing the incidence of renal failure.
